# A simple 3D cellular chemotaxis assay and analysis workflow suitable for a wide range of migrating cells

**DOI:** 10.1016/j.mex.2019.11.001

**Published:** 2019-11-16

**Authors:** Sai P. Visweshwaran, Tanja Maritzen

**Affiliations:** Leibniz-Forschungsinstitut für Molekulare Pharmakologie (FMP), Robert-Rössle-Straße 10, 13125 Berlin, Germany

**Keywords:** 3D cellular chemotaxis assay and analysis workflow, Migration, Motility, Collagen, Dendritic cells, Cancer, Directionality, Automated cell tracking, Cell speed, Mean Squared Displacement (MSD), Three dimensional cell migration, TrackMate, T cells

## Abstract

Cellular migration plays a crucial role within multicellular organisms enabling organ development, wound healing and efficient immune responses, but also metastasis. Therefore, it is crucial to dissect the underlying mechanisms. Directed migration and invasion are most efficient in response to chemotactic signals. To study cell migration and chemotactic responses, current experimental setups use either simplified 2D, tissue-mimetic 3D (e.g. collagen matrices) or *in vivo* environments. While the *in vivo* experiments are closest to the real physiological situation, they require animal experiments and are thus ill-suited for screening purposes. 3D matrices, on the other hand, can mimic *in vivo* conditions in many respects thus serving as instructive settings for the initial dissection of cell migration and chemotaxis. However, performing 3D chemotaxis assays has its limitations due to the delicate nature of most available setups and the tedious and time-consuming manual quantification process. Here, we present

• A method for the easy construction of a chemotaxis chamber suitable for the analysis of large cell numbers.
• A procedure to quantify their migration automatically with minimal input required by the experimenter.
• Both successfully validated by analyzing the 3D chemotaxis of highly migratory primary dendritic cells and the invasive MDA-MB-231 cancer cells.

## Graphical abstract

**Figure:**
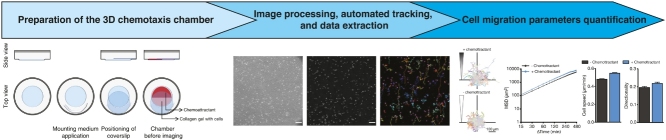

**Specification Table**

**Table:** 

Subject Area:	Biochemistry, Genetics and Molecular Biology
More specific subject area:	Cell Biology of Cell Migration
Method name:	3D Cellular Chemotaxis Assay and Analysis Workflow
Name and reference of original method:	M. Sixt, T. Lämmermann, In vitro analysis of chemotactic leukocyte migration in 3D environments, Methods Mol. Biol. 769 (2011) 149–165. doi:https://doi.org/10.1007/978-1-61779-207-6_11. B. Niggemann, S. Keil, N. Rommerswinkel, T. Dittmar, K.S. Zänker, Analysis of Cell Migration within a Three-dimensional Collagen Matrix, J. Vis. Exp. (2014) 1–10. doi:https://doi.org/10.3791/51963. V. Biswenger, N. Baumann, J. Jürschick, M. Häckl, C. Battle, J. Schwarz, E. Horn, R. Zantl, Characterization of EGF-guided MDA-MB-231 cell chemotaxis in vitro using a physiological and highly sensitive assay system, PLoS One. 13 (2018). doi:https://doi.org/10.1371/journal.pone.0203040.
Resource availability	Fiji - ImageJ: https://imagej.net/Fiji/Downloads

The ability to migrate efficiently is a hallmark of various cell types in our body and plays a crucial role in several physiological processes, including embryonic development, wound healing, and immune responses [[1], [2], [3]]. Defects in cell migration during development cause malformations, which can lead to the early death of the embryo or multiple syndromes including neurological disorders and congenital heart diseases [4]. In the context of our immune system, altered migration can cause serious conditions ranging from immunosuppression to autoimmune diseases. Unregulated migration can contribute to chronic inflammation syndromes such as asthma, psoriasis, rheumatoid arthritis, Crohn's disease, and multiple sclerosis. In these cases, immune cells migrate and become active at inappropriate sites causing tissue destruction [5]. The cell migration program can also be activated in normally non-motile cells, such as epithelial cells, during tumorigenesis, which allows them to invade the body to form metastases [6].

Although the last decade has witnessed enormous advances in our understanding of the mechanisms underlying the highly plastic process of cell migration, many questions remain open, especially regarding the complex regulation of directional migration [7,8]. Cells can migrate either randomly or directionally, with directional migration being paramount for efficient long-distance travel [[7], [8], [9], [10], [11]]. The directionality of cells is governed by their ability to recognize and respond to environmental cues. Based on the location and types of the cue, cells can engage different types of directed migration. Among those different types, chemotaxis, a process of directed cell migration towards a chemical gradient, is critically involved in many biological processes, including immune responses, development, and metastasis [12,13]. It allows cells to efficiently navigate their way through complex 3D environments that are crowded with other cells and diverse extracellular matrix components. Examples of cell types that heavily rely on chemotaxis are dendritic cells (DCs) and carcinoma cells [[14], [15], [16], [17]]. DCs are often called the sentinels of our immune system since they are stationed in peripheral organs and upon the encounter of pathogens travel to lymph nodes to alert T cells to fight off the infection [18,19]. The migration capacity of DCs and T cells determines the efficiency of our adaptive immune response, while the migratory abilities of cancer cells promote metastasis. Unraveling the underlying migration mechanism deployed by such cells is key for manipulating them effectively for therapeutic approaches [[20], [21], [22]].

To analyze cell migration, several *in vitro* and *in vivo* cell migration assays have been developed over the years. Although *in vivo* cell migration assays most closely reflect the physiological situation by observing cells within their natural environment with its complexities of variable extracellular matrix (ECM) composition, geometry, topography and pore size, performing such experiments is labor- and cost-intensive, time-consuming, tough to control and requires advanced imaging techniques and animal experiments. Due to such practical challenges, cell migration has traditionally been studied on two-dimensional (2D) surfaces [23] e.g. in the context of wound-healing assays [24]. While this works to some extent for adherent cells such as breast epithelial carcinoma cells, 2D migration assays have little physiological relevance and thus little predictive value for loosely or non-adherent cells such as DCs. In line with this notion, the chemotactic movement of DCs deficient for the small GTPase Cdc42 was only moderately impaired in 2D, while their *in vivo* migration was completely abolished. This strong migratory defect was far better predicted by directed migration assays in 3D collagen gels where the knockout cells displayed already strong decreases in speed and directional persistence [25]. The striking difference between the 2D and the 3D setting becomes understandable in the light of recent studies of cell motility [[26], [27], [28], [29], [30]] which demonstrate that cell migration is a very plastic process in which cells embedded in 3D matrices composed of collagens or matrigel employ a very different locomotory machinery than cells on 2D surfaces. Consequently, studying the migration of cells that are embedded within a 3D environment leads in most contexts to results that are more meaningful.

Apart from being easier to perform than true *in vivo* migration experiments, 3D migration assays with their simpler matrix composition offer the advantage of a controlled, easily manipulable environment which can facilitate the dissection of molecular mechanisms and the interpretation of experimental results. 3D migration, especially of non-adherent cells, has also been studied with the help of Boyden chambers (e.g. transwell assays [11,31]). However, these assays typically provide only an endpoint readout of cell migration efficiency, thereby strongly limiting the information that can be derived for the dissection of molecular mechanisms. In contrast, real-time microscopy based 3D assays allow the tracking of individual cells and thus the analysis of additional parameters such as speed and directionality.

However, many currently available methods for studying 3D cell migration have their limitations in that they either allow the experimenter only to analyze random 3D migration [9,11] since chemokine gradients cannot be established, or compel the experimenter to use complex, hard-to-handle and often costly setups [[32], [33], [34]] to perform 3D chemotactic migration assays. In addition, the quantification process in both scenarios has been tedious and time-consuming since it involved manual cell tracking. To overcome these limitations we have developed an easy method for performing and analyzing 3D chemotactic migration assays based on a home-made chemotaxis setup and an automated analysis pipeline. In this paper, we provide a detailed protocol for the construction, operation and data analysis of a 3D chemotaxis migration assay that is suitable for migratory cells ranging from primary murine DCs to highly invasive cancer cell lines such as MDA-MB-231 cells.

## Method details

### Materials

**Table:** 

Reagent	e.g. Company	Catalog Number	Comments
35 mm dish (μ-dish)	Ibidi	81158	Glass bottom
22 mm circular coverslips	Jena Bioscience	CSL-104	
Richard-Allan Scientific Cytoseal 60	ThermoFisher Scientific	8310-4	
10× MEM	Sigma-Aldrich	M-0275	
NaHCO_3_	Sigma-Aldrich	SB761	Conc.: 7.5%
Collagen I, bovine	Nutacon	5005-B	Conc.: 3 mg/ml
Recombinant murine CCL19	Peprotech	250-27B	Also known as MIP-3β
Recombinant human CXCL12	Sino Biological	10118-HNAE	Also known as SDF-1
Insert for 6 petri dishes (35 mm; clampable)	Pecon	800182	
CO_2_-Cover for the insert for 6 petri dishes	Pecon	800121	

### Procedure

#### Preparation of the 3D migration chamber

**Fig. 1:**
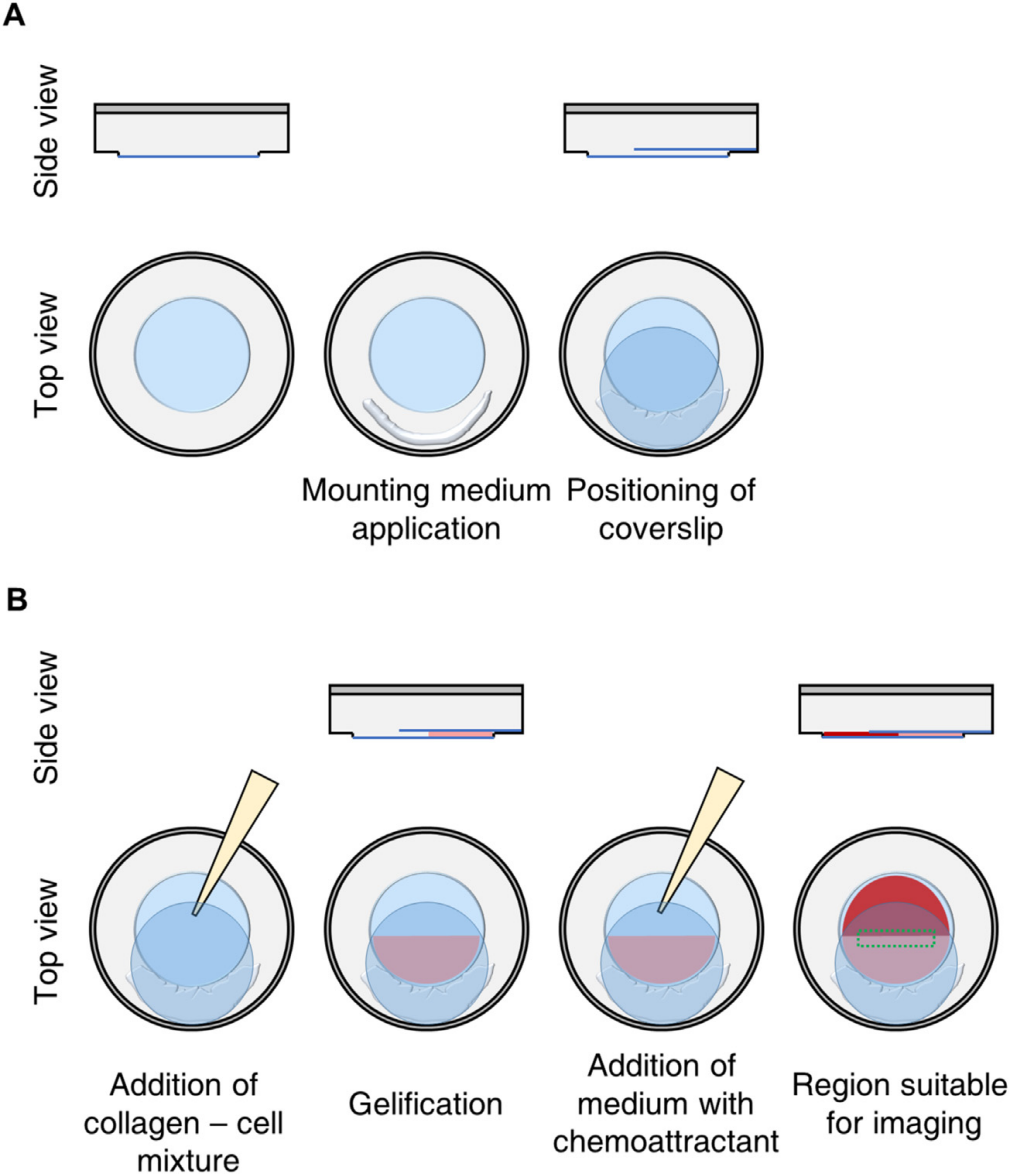
Steps in migration chamber assembly. Illustration of the steps necessary for building and filling the migration chamber. (**A**) In the depicted 35 mm petri dish, the central blue region represents the 21 mm wide round, ∼1 mm deep indentation with glass surface, which is surrounded by a plastic rim that is depicted in gray color. The second image shows where to pipette the mounting medium. The third image visualizes how to position the 22 mm coverslip in order to generate a pocket-like cavity. (**B**) The first image depicts where to add the collagen – cell mixture. The second image shows what the gel should look like after gentle tapping and gelification. The third image visualizes where to add the medium. The fourth image highlights the region of the gel that is recommended for imaging.
b To allow the mounting medium to dry, incubate the 35 mm dish for 30–60 min at room temperature inside the laminar airflow hood.

**NOTE:** Similar 35 mm glass-bottom dishes can be obtained from different manufacturers, important is the central indentation of about 1 mm depth. The chambers can be prepared in advance in a sterile environment and stored in a sterile manner for several days at room temperature for later use.

a Take a 35 mm plastic dish having at its center a 21 mm wide round ∼1 mm deep indentation with a glass bottom, which is surrounded by a plastic rim (e.g. available from Ibidi as 35 mm μ-dish). Inside a laminar airflow hood, pipette 10 μl of mounting medium onto one-half of the plastic rim surrounding the indentation. Immediately, take a sterile 22 mm circular glass coverslip using forceps and place it onto the half of the chamber previously covered with mounting medium in such a manner that the coverslip touches the wall of the chamber thus leaving part of the indentation uncovered, thereby creating a pocket-like cavity as shown in Fig. 1A.

#### Preparation of the collagen gel matrix embedded with cells

a Harvest the cells of interest and count them. Resuspend the cells in complete medium containing fetal bovine serum (FBS), antibiotics and the recommended supplements. The final cell concentration should be 0.5 × 10^6^ cells per ml. Keep the cells at room temperature and proceed quickly to step b.
b Prepare a collagen gel for each cell type by mixing the following ingredients as described below:

**Table:** 

Cell type	DCs	MDA-MB-231
Collagen gel concentration	1.4 mg/ml	1.6 mg/ml
1 10× MEM	15 μl	15 μl
2 NaHCO_3_	5 μl	5 μl
3 1 × MEM	35 μl	25 μl
4 Collagen I (Bovine) (3 mg/ml)	70 μl	80 μl
5 Cell suspension (0.5 × 10^6^cells/ml)	25 μl	25 μl
Total	150 μl	150 μl

Keep ingredients #1-4 on ice. First, pipette ingredients #1-3 into a 1.5 ml Eppendorf tube, mix gently by pipetting and keep on ice. Take the required volume of collagen I and add to the #1-3 mixture. Mix homogeneously by pipetting very gently without making any bubbles. As the last step add the cell suspension and again mix gently by pipetting.

c Take the prepared migration chamber and hold it vertical. Pipette 130 μl of the collagen gel – cell mixture gently into the pocket-like cavity as shown in Fig. 1A.
d Hold the 35 mm dish vertically and gently tap it a couple of times against the bench surface so that the gel completely settles down at the bottom of the cavity and forms a meniscus, which is horizontally straight and even (Fig. 1B).
e Place the dish horizontally again and allow the suspended cells to settle evenly.
f Put on the lid of the 35 mm dish making use of its locking feature to prevent drying out. Keep the dish in the cell culture incubator at 37 °C and 5% CO_2_ for 30–60 min for gelation, wait until the collagen gel becomes slightly milky white and collagen fibers become uniformly visible.

**NOTE:** This protocol assumes that the experimenter is familiar with the culture of bone marrow derived DCs and MDA-MB-231 cells. Information on these cell culturing techniques is already published [11,31]. The quality of the cells has to be ascertained prior to the experiment. Differentiation and maturation of DCs are routinely checked by flow cytometry analysis of differentiation and maturation markers as described in [31]. Where necessary, cell viability should be confirmed prior to the migration assay using approaches like Annexin V or PI staining as described in [35].

Other types of gels (e.g. matrigel, hydrogels) and gel compositions can also be used in this setup. The choice of matrix and the chosen density is a decisive factor for each 3D migration experiment and has to be carefully considered in line with the objectives of the experiment since it will directly affect migration parameters. For DCs 1.1–2.3 mg/ml are a usual range of concentration for collagen [30]. The higher the chosen collagen concentration is, the denser the resulting collagen fiber matrix will be. While a certain concentration is necessary to constitute a fiber meshwork, a further increase in the concentration and therefore the density of the matrix will decrease the cell migration speed. Thus 1.1 mg/ml collagen supports the fastest DC migration, while a matrix based on a 2.3 mg/ml collagen concentration will considerably slow down the average DC migration speed [30]. For cancer cells, the density also influences their mode of migration. Cancer cells navigating through densely packed collagen, for instance within a tumor, use invadopodia and matrix metalloproteinase activity to move, while cells in regions with less dense collagen and long, aligned fibers were reported to migrate rapidly using larger pseudopodial protrusions or MMP-independent amoeboid blebbing [36,37]. Thus, the exact type of matrix used will have a large impact on the result and should be well considered by the investigator depending on the cell type and the specific goals of the experiment. Certain impairments of hem1 knockout mDCs were, for instance, more apparent using a higher collagen concentration [30].

#### Time-lapse video acquisition

a During the gelation of the collagen gel, switch on the microscope and the microscope stage heater. Adjust the temperature to 37 °C.
b After gelation is complete, fill up the migration chamber gently with (about 240 μl) chemoattractant-free or chemoattractant-containing medium (Fig. 1B).
c Place the 35 mm dish into a microscope insert designed to hold 6 × 35 mm petri dishes and allowing for temperature, humidity, and CO_2_ control. Place the insert onto the microscope stage.
d Set the focus on the cells that are in the peripheral region of the gel adjacent to the interphase between gel and medium as shown in Fig. 1B.
e Use a 4× objective or a 4× objective combined with 1.5× zoom or a 10× objective. Use an objective with phase contrast.
f Acquire time-lapse images: DCs: 4 h with a time-lapse interval of 2 min. MDA-MB-231: 24−30 h with time-lapse interval of 15 min. Supplemental video 1 and 2 show examples of DCs resp. MDA-MB-231 cells moving in the collagen gel.

**NOTE:** The minimum chemoattractant concentration that is optimal for the cell type studied should be determined to improve the chemotactic efficiency of the cells of interest. If necessary, the chemoattractant gradient can be improved by first filling the chamber with just medium and then adding a small drop of chemoattractant (2–5 μl) to it, just before starting the image acquisition. In this manner, the chemoattractant first diffuses within the medium and then towards the gel, forming an extended gradient. If no phase contrast setup is available, cells can be fluorescently labeled using dyes such as TAMRA, and fluorescent time-lapse imaging can be performed.

#### Image processing

**Fig. 2:**
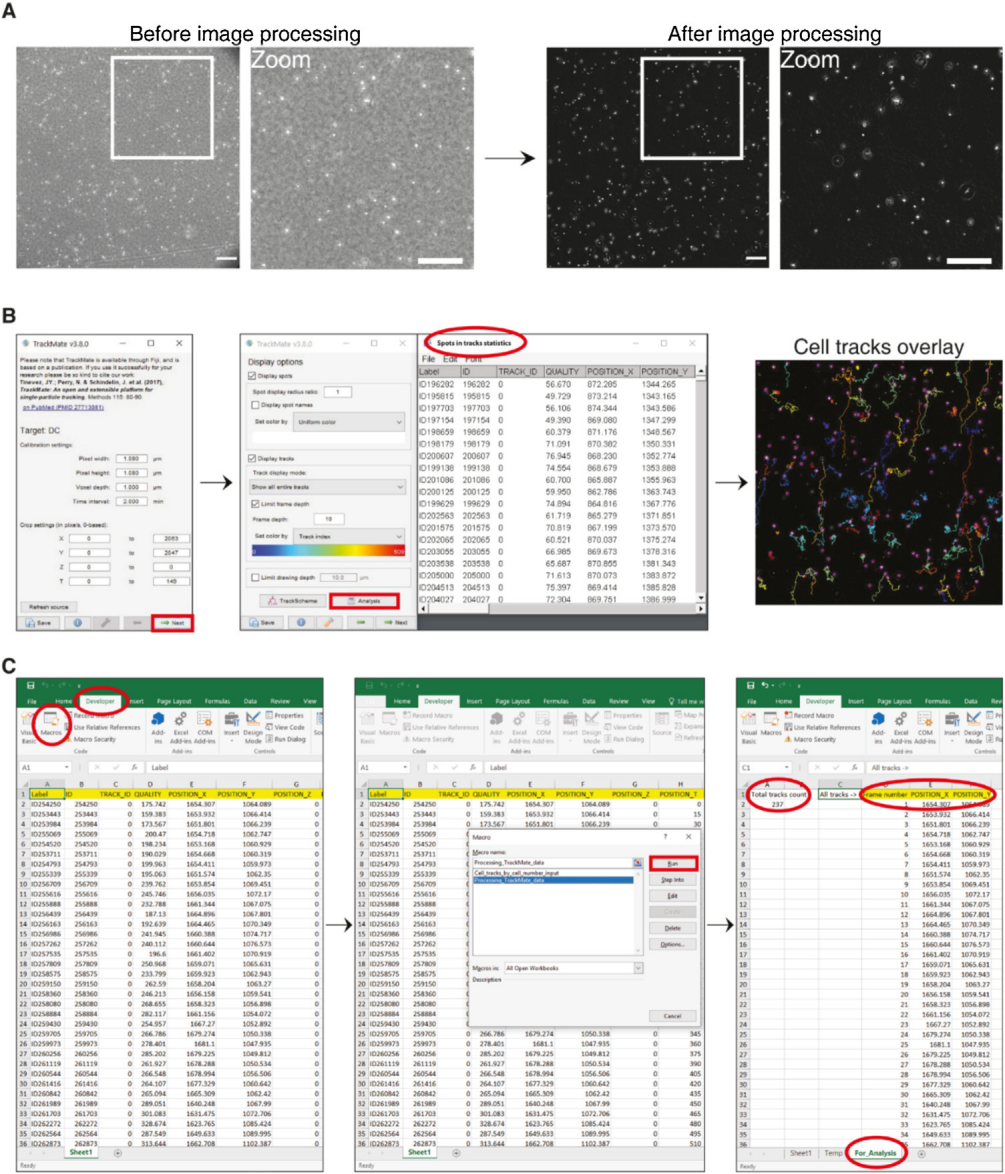
Image processing and automated tracking. (**A**) Representation of a phase-contrast image of the collagen gel containing mature DCs, before and after image processing to produce background-subtracted images. Scale bar: 200 μm. (**B**) Snapshots of the TrackMate plugin from Fiji to track the cells. The buttons and pop-up window that are to be noticed as described in the protocol are highlighted in green. (**C**) Snapshots of the supplementary file 2 (MS Excel file) TrackMate_data, sheet1 containing the trajectories data that is copied from TrackMate - 'Spots in tracks statistics' for data extraction. By running 'Processing_TrackMate_data' macros, a sheet called 'For_Analysis' is generated, which contains the number of total tracks ('Total tracks count'), 'Frame number', 'Position_X' and 'Position_Y' columns that are used further to compute the migration parameters such as MSD, cell speed, and directionality. Screen captures of all the steps involved are provided in supplemental video 3.

The image processing and quantification steps are depicted briefly in Fig. 2. Detailed screen captures of all steps are provided in supplemental video 3.

**Assigning the image properties:**

Open the video/image stack file in Fiji (in the supplement we provide with video 4 and video 5 two files to test the analysis pipeline with) and assign the image properties by selecting ‘Image’ → ‘Properties…’ and fill in ‘Unit of length’ (example - μm), ‘Pixel width’ (pixel size in μm), ‘Pixel height’ (pixel size in μm) and ‘Frame interval’ (example for DCs - 2 min). Save the video/image stack. A similar assignment could also be done by selecting ‘Analyze’ → ‘Set scale’ option.

**Automated processing**:

a To enable the automated tracking program to clearly recognize the cells, we developed image-processing steps for phase-contrast images. These image-processing steps comprise: creating a Z projection of the stack images, performing an image subtraction with it and a contrast enhancement. A detailed description is given below in the manual processing section. To perform this as batch image processing we have created a macro (see **Supplemental File 1: 3D_migration_image_processing**), which is written in Java.
b Before running the macro, create two folders in the directory where the folder containing the 3D migration videos is located. Run the macro in Fiji by going to ‘Plugins’ → ‘Macros’ → ‘Run’, then select the **3D_migration_image_processing** file. The program will ask you to choose an input folder. Select the folder containing your 3D migration videos. Then the program will ask you to choose a folder for saving the Z projections of the videos. For this, select one of the newly created folders in the same directory where the input folder is located. Finally, the program will ask you to choose a folder for saving the final background-subtracted images. Select the other newly created folder. After execution, the macro will generate the final background-subtracted images that are ready for automated tracking.

**Alternative manual processing**:

a **Create a Z projection of average intensity**

Open the properties assigned video/image stack file in Fiji. Go to ‘Image’ → ‘Stacks’ → ‘Z Project…’ → select ‘Start slice: 1′, ‘Stop slice: last frame number’ and ‘Projection type: Average Intensity’. Save the generated file. For example, let us assume the video1 was processed as above to generate a Z-projection and it is stored as ‘video1_Zprojection’.

b **Background subtraction and contrast enhancement**
c Go to ‘Process’ → ‘Image Calculator…’ → select ‘Image 1: current video/stack image name (e.g. video1)’, ‘Operation: Subtract’, ‘Image 2: Z-projection of the same file (e.g. video1_Zprojection)’ and execute by clicking ‘Ok’. A background-subtracted image file will be generated.
d To enhance the contrast of the background-subtracted file, go to ‘Process’ → ‘Enhance Contrast…’ → assign ‘Saturated pixels: 0.1 %’, ‘Normalize: Yes‘, ‘Equalize histogram: No’, ‘Process all n slices: Yes’, ‘Use stack histogram: No’.
e Save the file.

**NOTE:** The input folder should only contain videos files/image stack files. The folders created to store Z projection and final background-subtracted images should not be inside of the input folder. They should be separate individual folders located in the same directory as the input folder. This processing is meant for phase-contrast images. For fluorescent images where cell-tracking dyes were used, improving the contrast of the videos as described above might be sufficient.

#### Automated cell tracking and extraction of the cell track data

For cell tracking, we used TrackMate (version 3.8.0), an open and extendable platform for single-particle tracking. This is a plug-in available in the Fiji software bundle [38]. It offers a versatile and modular solution for end-users through a simple and intuitive user interface. Here, in our method, we utilized its particle tracking ability in 2D images. The cell track data generated by the TrackMate tool should be copied to **supplemental file 2 (MS Excel): TrackMate_data.xlsm, sheet1** for further analysis as explained below.

**Steps for cell tracking with TrackMate**:

a Open the background-subtracted 3D migration video in Fiji.
b Go to ‘Plugins’ → ‘Tracking’ → ‘TrackMate’ → Check the image properties, if all fine → Next
c For DCs: Select ‘LoG Detector’ → ‘Estimated blob diameter: 30 μm’, ‘Threshold: 25′, ‘Use median filter: No’, ‘Do sub-pixel localization: Yes ‘→ Next

For MDA-MD-231: Select ‘LoG Detector’ → ‘Estimated blob diameter: 40 μm’, ‘Threshold: 25′, ‘Use median filter: No’, ‘Do sub-pixel localization: Yes ‘→ Next

d Software runs to recognize the cells → Next
e ‘Initial thresholding: Quality - Auto’ → Next
f ‘Select view: Hyperstack Display’ → Next
g ‘Set filters on spots: No selection’ and ‘Set color by: Uniform color’ → Next
h Select: ‘Simple LAP tracker’ → Next
i ‘Simple LAP tracker’: ‘Linking max distance: 50 μm’, ‘Gap-closing max distance: 50 μm’, ‘Gap-closing max frame gap: 8′ → Next
j Software runs to process the cell tracking → Next
k Set filter on tracks: click (+) button and select ‘Maximal quality: Auto’ and again click (+) button and select ‘Duration of tracks: Auto’. Keep’ Set color by: Track index’, click → Next
l Keep ‘Display spot’, ‘Display tracks’ and’ Limit frame depth’ enabled/checked and the rest unchecked. Click ‘Analysis’, three pop-up windows will open. In that, copy the data of the 'Spots in tracks statistics' window to supplementary excel file TrackMate_data, sheet1. This file contains the track information. Save the file. Click → Next
m Skip the ‘Spots’, ‘Links’ and ‘Tracks’ section and click → Next
n Select: ‘Capture overlay’ → Execute – This will produce a video with tracks overlaid. Save the file.

**Extraction of the data**

a The TrackMate data of cell tracks has to be extracted and processed for further analysis. For this, we have created **macros that are preloaded in the TrackMate_data MS Excel file**.
b To run the macro go to ‘Developer’ → ‘Macros’ → select ‘Processing_TrackMate_data’
c This is the main program that will generate a ‘Temp’ sheet and a ‘For_Analysis’ sheet. The final migration trajectory data will be in the ‘For_Analysis’ sheet column ‘D’, ‘E’ and ‘F’, as well the total number of tracks generated will be shown in ‘A’ column, second row.
d As a sub-option, to extract a user-preferred number of cell tracks from the total number of tracks, run the macro ‘Cell_tracks_by_cell_number_input’ which is also contained in the TrackMate_data file. The program will generate a user-defined number of tracks in column ‘J’, ‘K’ and ‘L’.

**NOTE:** The TrackMate parameter ‘Estimated blob diameter’ and ‘Threshold’ in step 3 varies with the cell type and objective. The user needs to determine the blob diameter and threshold that is optimal for the detection of his/her cell type with less background by using the ‘Preview’ option. For a 4x objective with 1.5x zoom video acquisitions, TrackMate cell tracking will yield 100–150 cell tracks per video. If the TrackMate plugin is not able to recognize the cells, increasing the contrast of the video might improve the tracking efficiency.

For highly motile cells, cell division is unlikely to affect the analysis due to the comparably short tracking time, however, with tracking intervals >24 h, the likelihood increases that a fraction of the analyzed cells undergoes cell division. Still, even with longer tracking intervals we hardly observed any cell division events for the MDA-MB-231 cells. Besides, if a cell division event should occur during the tracking interval, the TrackMate setting “Simple LAP tracker”, which we advise in our protocol to select for tracking, precludes the detection of splitting and merging events [38]. Consequently, the dividing of a tracked cell will cause the tracking algorithm to abort the tracking of this cell. Thus, it is normally not necessary to remove cell division events from the data.

For running the macros in MS Excel, ‘Developer’ tab has to be enabled by going to ‘Excel Options’ → ‘Customize Ribbon’ → ‘Customize the Ribbon’ → ‘Main tab’ → checking ‘Developer’. Macro ‘Processing_TrackMate_data’ has to be run first, and then only the user can run the ‘Cell_tracks_by_cell_number_input’ macro.

#### Quantification of cell motility parameters

To analyze cell tracks and compute parameters like cell trajectories, mean squared displacement (MSD), cell speed and directionality we used DiPer, the custom-made open-source suite of a computer program created by Gorelik & Gautreau [39]. This user-friendly program is executable through Microsoft Excel, and it generates plots of publication-level quality. We made minor improvements to the source code of this program to enable it to quantify high volumes of data. The Microsoft Excel file containing these macros is available as **supplemental file 3: Track_quantification.**

**A detailed procedure and troubleshooting can be found in the original paper** [39]. **Briefly:**

a Arrange your migration trajectory data according to the following format: Each worksheet in the **supplemental file 3: Track_quantification** must correspond to one condition. For example, if you have one control condition and two experimental conditions, your file should contain three labeled worksheets (e.g., control 1, sample 1 and sample 2). To label worksheets, double-click on the worksheet tab (at the bottom of the screen) and type in the name.
b Copy the migration trajectory data from the previously generated **TrackMate_data excel file.** Paste the data (Frame number, Position_X and Position_Y) to the columns ‘D’, ‘E’ and ‘F’ of **Track_quantification**.
c Each worksheet must contain all trajectories for that condition - listed one after the other without any gaps. So do not insert empty rows between trajectories. If your excel file contains empty worksheets, delete them by right-clicking and selecting ‘Delete’.
d Save your file under a new name, such as ‘Exp 1.xlsm’. We suggest keeping this original file as it is and not running any programs on it. We refer to this file as the ‘Original File’.
e Save the Original File for a second time under a new name. We refer to this second file as the ‘Copy File’. You will run programs on the Copy File.
f To run the various programs/macros to compute cell migration parameters, click on the Developer tab.
g Click the Macros button.
h Select a program from the list that will appear (e.g. Speed, MSD etc.).
i Press ‘Run’. The status bar at the bottom left of the screen will display ‘Please wait …calculations in progress’. When the program finishes, the status bar will display ‘Ready’.
j Some programs will ask you to input parameters, such as the time interval between frames. Please type only numbers and omit the units. (The latter can be typed into the axes labels of the graphs.) Press ‘OK’ or ‘Enter’.
k Your results will now be displayed in Microsoft Excel. Save the resulting file under a new name. We suggest appending the name of your file with the name of the program, for example, ‘Exp1_MSD.xlsm’.

**NOTE:** The Plot_At_Origin macro in the **Track_quantification excel file,** that draws the trajectories of the cells, has a limitation. As a chart in MS Excel can only contain up to 256 series, only 256 cell trajectories per condition can be plotted. So to plot cell tracks, run Plot_At_Origin with trajectory information for 10–150 cells for optimal performance of the macro and graphical representation. If users have more than 256 tracks, then they should run the ‘Cell_tracks_by_cell_number_input’ macro preloaded in the **TrackMate_data MS Excel file**, which will allow the user to extract less than 256 cell tracks, which then can be used to run the Plot_At_Origin macro in the **Track_quantification excel file.**

### Method validation

**Fig. 3:**
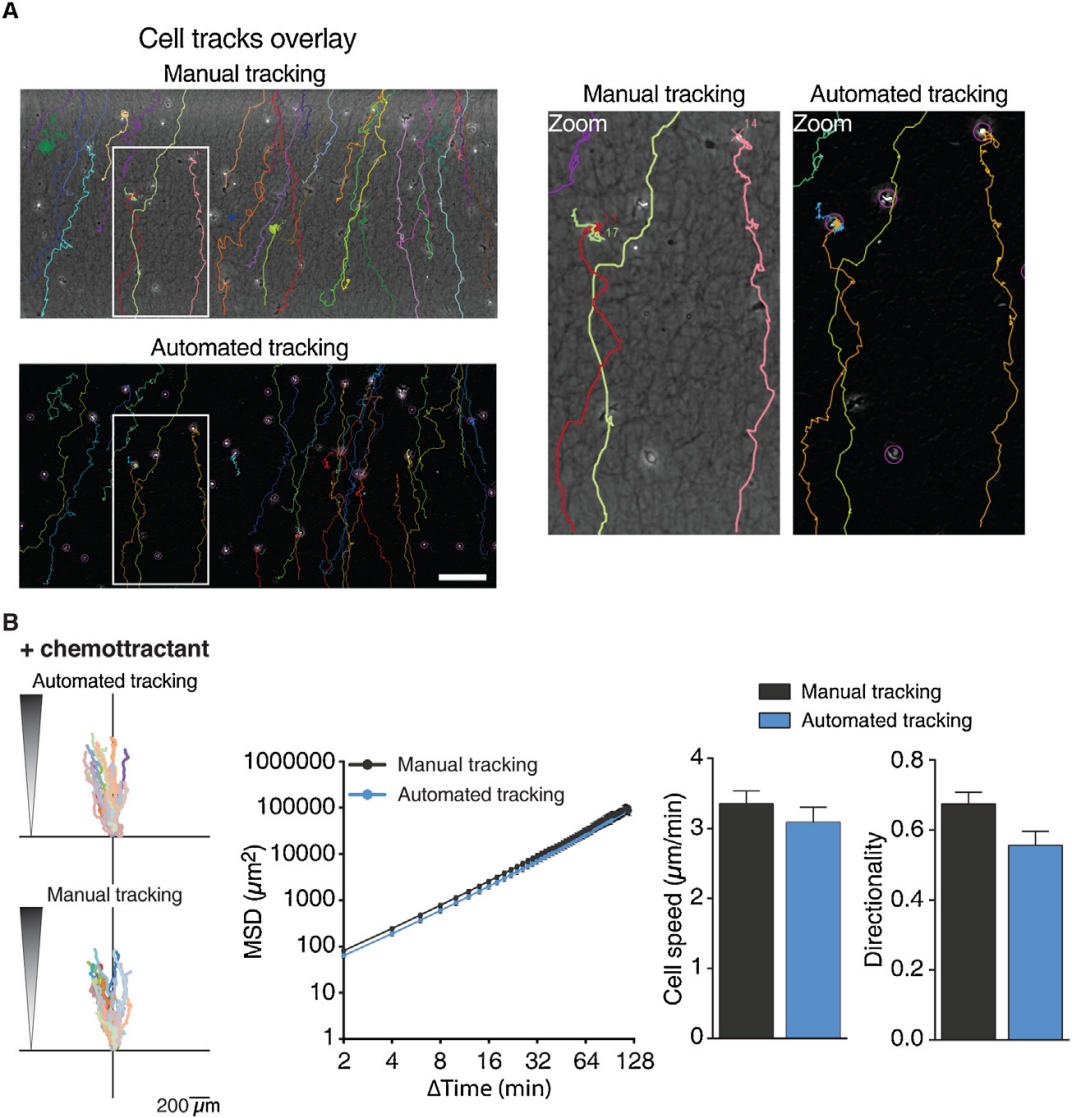
Comparison of manual and automated cell tracking. 3D chemotaxis of mDCs towards a chemoattractant gradient was quantified (650 ng/ml CCL19 was used). (**A**) Overlay of microscopy images (after image processing in case of automated tracking procedure) and cell tracks derived from either manual or automated tracking. Scale bar: 200 μm. Zooms of white boxes are depicted to the right. (**B**) Left: 3D single-cell trajectories in the presence of chemokine following either automated or manual cell tracking. Middle: Mean squared displacement (MSD) with MSD and time intervals displayed on log scales. Right: Quantification of cell speed and directionality. 30 cells were analyzed by manual and automated tracking. Data are mean ± SEM.

An essential step in the validation of our automated cell tracking approach was to visually inspect the obtained tracks to confirm that we follow real cell movements and do not track artefacts. For this, we carefully looked at the files generated by TrackMate’s ‘Capture overlay’ function that produces videos with overlaid tracks. Our inspection in fact validated the cell tracks generated by TrackMate (Fig. 3A, and compare also track depiction in Fig. 2B) and did not reveal any artefacts.

Another critical point was the comparison of results obtained for the same data set by manual and automated cell tracking. We performed this analysis with an exemplary migration video of mature DCs migrating towards chemoattractant (Fig. 3). We tracked a sample population of 30 cells using on the one hand manual tracking and on the other hand our automated tracking approach. As expected, the tracks generated by both methods look very similar, and the quantified migration parameters are consequently also very similar, however, not identical (Fig. 3B). The obtained mean squared displacement (MSD), which measures the area explored by the cell over time, is nearly identical between the two data sets, and also the error bars for the cell speed are overlapping. However, there is a slight variation in regards to the quantified directionality. When comparing the tracks that were generated for the same cell by manual and automated tracking in detail, it is indeed noticeable that the manually obtained cell tracks appear straightened out when compared to the tracks generated by TrackMate (Fig. 3A, Zoom). Reasons for this observation were already discussed by Gorelik and Gautreau: Manual tracking is susceptible to the “grid effect” which leads to an overrepresentation of certain angles between displacements (such as 0, 45, 90, 135, 180). In addition, when a cell remains in place from one frame to the next, the user is prone to click on the same pixel again producing repeat coordinates which does not happen in automated tracking [39]. Besides, the repetitive and fatiguing task of manual tracking also promotes clicking on the same spot again while following the cell which increases the likelihood of straightened out and thus a bit shorter tracks. As directionality is the ratio of displacement over trajectory length, the smoothed out trajectories obtained by manual tracking are expected to result in a slightly higher value of directionality compared to automated tracking as we observe it in our experiment. To allow a direct comparison, we only quantified 30 cells here for both approaches. However, a major reason why automated tracking is expected to yield in the end more reliable results than manual tracking is the much higher cell number that can easily be quantified (more than 100 cells for a condition) leading to more representative mean values for cell migration parameters.

**Fig. 4:**
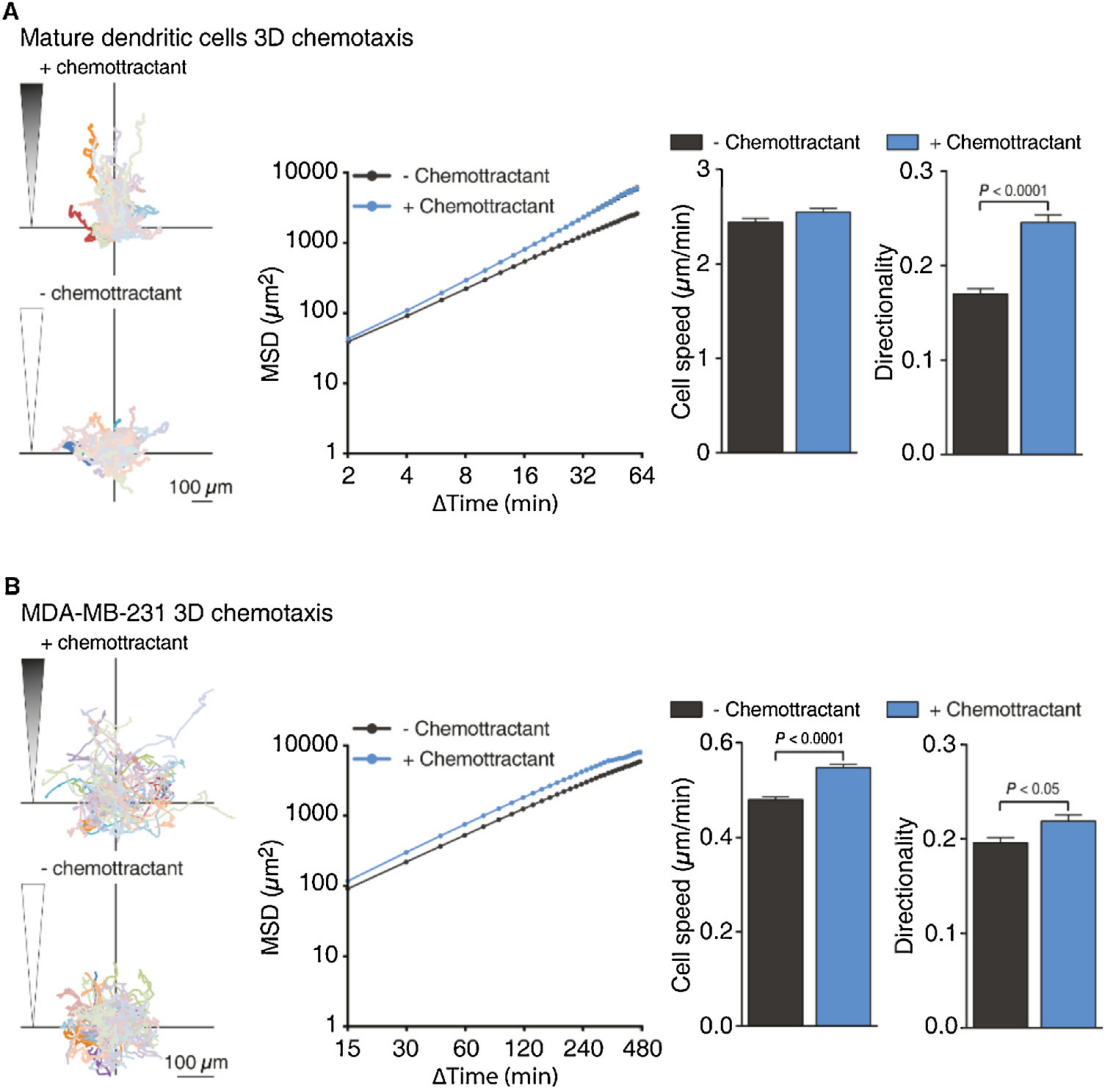
Exemplary results obtained with DCs and MDA-MB-231 cells. Mature DCs (mDCs) (**A**) and MDA-MB-231 cells (**B**) were probed for 3D chemotaxis. As chemoattractant CCL19 (650 ng/ml) was used for DCs and CXCL12 (25 ng/ml) for MDA-MB-231 cells. Left: 3D single-cell trajectories in the presence and absence of chemokine. Middle: Mean squared displacement (MSD) with MSD and time intervals displayed on log scales. Right: Bar diagrams for cell speed and directional persistence. In the presence of chemokine, there is a significant increase in directionality for mDCs and MDA-MB-231 cells. For DCs, cells were harvested from three independent cultures of murine wild-type bone-marrow-derived DCs. For MDA-MB-231, cells were harvested from two independent cultures. A minimum of 100 cells per condition was tracked for each experiment. For the quantification all tracked cells from all independent experiments were pooled. Data are mean ± SEM. Statistical significance was probed with a Mann-Whitney test. Screen captures of all the steps involved in the quantification process are provided in supplemental video 3.

Having established the reliability of the automated cell tracking approach, we used our cell migration analysis workflow to compare migration parameters of mature DCs and MDA-MB-231 cells in the presence or absence of a suitable chemoattractant which is CCL19 (650 ng/ml) for DCs and CXCL12 (25 ng/ml) for MDA-MB-231 cells. The cells were not starved, but directly subjected to the experiment. For imaging, we chose an area close to the region of the gel that touches the chemokine-containing medium as shown in Fig. 1B. After video microscopy, we proceeded with image processing as outlined in the protocol section. The results of our analysis are depicted in Fig. 4. Exemplary video montages of DC and MDA-MB-231 3D chemotaxis are available as supplemental videos 1 and 2.

The trajectory plots of individual cells visualize that mature DCs and MDA-MB-231 cells move randomly in the absence of a chemoattractant gradient. However, in the presence of chemokine, both cell types migrate directionally along the chemoattractant gradient. In line with the known striking capabilities of mature DCs for long-range chemokine directed migration, their increase in directionality upon chemoattractant addition is much more striking than for MDA-MB-231 cells. At the same time, the highly migration-competent mature DCs do not further increase their already high migration speed upon chemokine, while the cancer cells do not only increase their directionality, but also become significantly faster upon encounter of chemoattractant. The increased directionality of the mature DCs, even though paired with an unaltered cell speed, causes them over time to explore a larger area in the presence of chemokine. This fact is reflected in the computation of the mean squared displacement (MSD). In case of the MDA-MB-231 cells the simultaneous increase in speed and a moderate increase in directionality results in a constant increase in MSD over time.

An important parameter to consider when setting up the experiment is the length of the analyzed video, especially for slow-moving cells since for recording a chemotactic response it is important that the chemokine gradient persists during the analyzed interval. Optimally, the persistence of the gradient should be evaluated. Alternatively, different lengths of the videos taken for a specific cell type/chemokine pair could be analyzed to monitor if an originally detectable chemotactic response disappears over time.

Overall, this example demonstrates the potential of our technique, which allows the quantification of a large number of cells from a single experiment, generating various parameters of cell migration to dissect the underlying molecular mechanisms. The assay can also easily be used to detect differences induced by drug treatments. This can be extended to the use of siRNA and the screening of molecules involved in the control of chemotactic or random migration.

## Discussion

Currently available microscopy-based 3D chemotaxis migration setups are hard to build and to handle, and it is difficult to control their physical properties such as gel thickness from one experiment to another [32,33]. The commercially available products are mostly based on the seeding of cells into microchannels [34]. Two of their drawbacks are the high possibility of having air bubbles in the observation chamber and the chances of rupturing the collagen gel by pipette action. This complicates their use especially for new experimenters and raises costs due to spoilt experiments. Here, we describe a robust method for performing hassle-free chemotaxis assays and provide an easy-to-adopt procedure for their automated quantification that is suitable for different cell types (e.g. T cells, neutrophils). The simple setup we designed based on a 35 mm dish with indentation (e.g. the Ibidi μ-dish) is suitable for live-cell imaging and super-resolution microscopy applications. In addition, the lid-lock design of the 35 mm μ-dish prevents evaporation of the medium, which allows 20–30 h long live-cell imaging without cells drying out.

Traditionally, live imaging 3D migration assays were quantified by manual cell tracking due to the interference of the out-of-focus cells that are located in different Z planes of the collagen gel, which makes it difficult for automated tracking software to reliably recognize individual cells. This holds especially true for experiments involving fast migrating cells like immune cells, which are more difficult to track due to their speed than slow-moving cells such as cancer cells. Due to the tediousness of manual tracking, it restricts the scale and scope of possible 3D migration assays. In our setup, the cast collagen gel will have a thickness of about 1 mm leading to a sufficiently thick matrix for 3D cell migration. At the same time, in combination with an optimized cell concentration in the collagen suspension, the gel is thin enough to achieve a distribution of cells in a single plane. Due to this, in our setup there are no out-of-focus cells, and the video looks two-dimensional even though the cells are migrating in a 3D matrix. This makes our setup highly suitable for analysis with automated tracking software. Furthermore, in combination with a microscopy holder for 6 × 35 mm dishes and an automated microscope stage that can be programmed to image different locations, our experimental system can be adapted to acquire as many as six different conditions in a single session.

Some of the critical steps in this protocol are: (1) to use an optimal cell count to suspend in the collagen gel (see collagen gel recipe table), (2) not to incubate the gel too long for the gelification process, (3) to avoid creating bubbles while making the gel. However, even when extra care is taken, there are sometimes bubbles present in the gel after gelification. Nevertheless, usually, in our setup they do not disturb the experiment since one can easily find bubble-free imaging areas due to the comparably large size of the medium-gel interface. (4) The optimal chemokine concentration has to be determined, as high concentrations might diffuse fast leading to a very shallow or no gradient. (5) The imaging area should be chosen close to the region of the gel that touches the chemokine-containing medium. Cells located very distantly from this region might behave differently due to the delayed arrival of the chemokine, thus keeping the imaging region constant for each experiment is an important factor for achieving high reproducibility.

In summary, our experimental system mimics the complex 3D environmental constraints found in tissues by endogenous migratory cells and allows the evaluation of chemotactic responses. It is easy to set up and allows for semi-automated analysis thus enabling the user to perform 3D chemotaxis or also random migration assays with different cell types with very little hands-on time.

Especially the automated tracking and analysis pipeline will likely also prove useful in other experimental systems. For example, it could also be applied to related 2D migration assays. The main difference here is that the phase contrast of cells on a 2D surface (e.g. a petri dish bottom coated with fibronectin) compared to cells in a 3D matrix is often much less pronounced. Therefore, under such conditions, it becomes mandatory to use fluorescent dyes for coloring the migrating cells to be able to identify them with sufficient reliability during the tracking. In addition, with adequate adjustments the tracking pipeline might also be adapted for the tracking of smaller objects like lysosomes or focal adhesions. The only important prerequisite is that the particle size should not change within the experiment since the tracking is set for a certain particle size in order to pick up the correct structures.

## Declaration of Competing Interest

The authors have no conflicts of interest to disclose.
