# Supplementary material for: A simple 3D cellular chemotaxis assay and analysis workflow suitable for a wide range of migrating cells
Source: MethodsX. 2019 Nov 16;6:2807–21. doi: 10.1016/j.mex.2019.11.001 (PMC6909357; doi:10.1016/j.mex.2019.11.001)
Supplement: Supplementary file 1 [file mmc1.docx]

**Supplementary material**

**Supplemental file 1. 3D Migration Image Processing**

**Supplemental file 3. TrackMate Data**

**Supplemental file 2. Track Quantification**

**Supplemental video 1. Mature DC chemotaxis in 3D collagen gel**

Cells were embedded in the collagen gel, and their migration was observed in the presence or absence of CCL19 (650 ng/ml). Cells were imaged for 5 h with pictures taken every 4 min using phase contrast and a 4x objective with 1.5x zoom. Scale bar: 200 µm.

**Supplemental video 2. MDA-MB-231 chemotaxis in 3D collagen gel**

Cells were embedded in the collagen gel, and their migration was observed in the presence or absence of CXCL12 (50 ng/ml). Cells were imaged for 30 h with pictures taken every 15 min using phase contrast and a 4x objective with 1.5x zoom. For depiction, the video has been cropped after 6 h. Scale bar: 200 µm.

**Supplemental video 3. Screen capture of the steps from image processing up to quantification**

An exemplary illustration of the steps of phase-contrast video processing, automated tracking with TrackMate, cell tracks data extraction, and quantifications of migration parameters. One of the MDA-MB-231 cell chemotaxis videos has been depicted as the example.

**Supplemental video 4. MDA-MB-231 cells in absence of CXCL12 - movie for testing the analysis pipeline**

Cells were embedded in the collagen gel, and migration was observed in the absence chemokine. This video is meant to be used for hands-on testing of the described analysis pipeline.

**Supplemental video 5. MDA-MB-231 cells in presence of CXCL12 - movie for testing the analysis pipeline**

Cells were embedded in the collagen gel, and migration was observed in the presence of 50 ng/ml CXCL12. This video is meant to be used for hands-on testing of the described analysis pipeline.
