# Supplementary figures and images for: A simple 3D cellular chemotaxis assay and analysis workflow suitable for a wide range of migrating cells
Source: MethodsX. 2019 Nov 16;6:2807–21. doi: 10.1016/j.mex.2019.11.001 (PMC6909357; doi:10.1016/j.mex.2019.11.001)

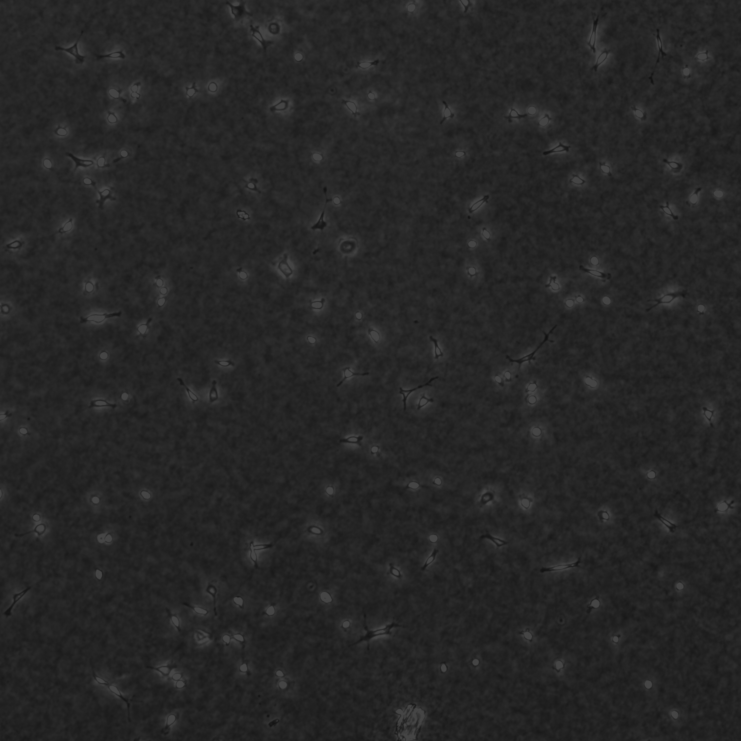

Supplement: Supplementary file 8 [file mmc8.zip › Sup_video5_test_with_plus_CXCL12_V3.tif]

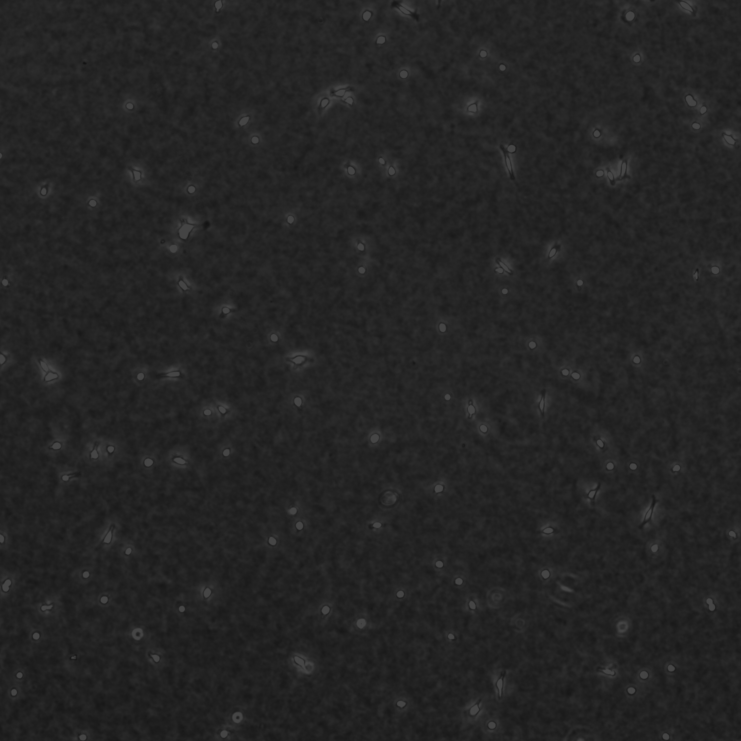

Supplement: Supplementary file 9 [file mmc9.zip › Sup_video4_test_with_minus_CXCL12_V3.tif]
